# Aging-Related Cellular, Structural and Functional Changes in the Lymph Nodes: A Significant Component of Immunosenescence? An Overview

**DOI:** 10.3390/cells10113148

**Published:** 2021-11-12

**Authors:** Marta Cakala-Jakimowicz, Paulina Kolodziej-Wojnar, Monika Puzianowska-Kuznicka

**Affiliations:** 1 Department of Human Epigenetics, Mossakowski Medical Research Institute, Polish Academy of Sciences, 02-106 Warsaw, Poland; pkolodziej@imdik.pan.pl; 2 Department of Geriatrics and Gerontology, Medical Centre of Postgraduate Education, 01-813 Warsaw, Poland

**Keywords:** aging, immunosenescence, lymph nodes, stromal cells, lymphatic endothelial cells, lymphocytes, neutrophils

## Abstract

Aging affects all tissues and organs. Aging of the immune system results in the severe disruption of its functions, leading to an increased susceptibility to infections, an increase in autoimmune disorders and cancer incidence, and a decreased response to vaccines. Lymph nodes are precisely organized structures of the peripheral lymphoid organs and are the key sites coordinating innate and long-term adaptive immune responses to external antigens and vaccines. They are also involved in immune tolerance. The aging of lymph nodes results in decreased cell transport to and within the nodes, a disturbance in the structure and organization of nodal zones, incorrect location of individual immune cell types and impaired intercellular interactions, as well as changes in the production of adequate amounts of chemokines and cytokines necessary for immune cell proliferation, survival and function, impaired naïve T- and B-cell homeostasis, and a diminished long-term humoral response. Understanding the causes of these stromal and lymphoid microenvironment changes in the lymph nodes that cause the aging-related dysfunction of the immune system can help to improve long-term immune responses and the effectiveness of vaccines in the elderly.

## 1. Introduction

Aging is an inevitable biological phenomenon. Even during healthy aging, the functions of the immune system may be weakened by a process known as immunosenescence [1]. Immunosenescence and inflammaging are responsible for the increasing incidence of infections, autoimmune diseases, and neoplasms in the population over 65 [2,3]. Older adults also show weaker responses to vaccination than younger ones. Aging causes adverse changes in the innate and adaptive parts of the immune system, the microenvironment of lymphoid organs where immune cells develop and reside, and the equilibrium of soluble chemokines and cytokines, all responsible for the functioning and homeostasis of the immune system [4]. Aging-associated changes in the primary lymphoid organs, i.e., bone marrow [5,6,7,8,9,10] and thymus [3,11], have been thoroughly characterized; however, data on aging of the secondary lymphoid organs, e.g., lymph nodes, is still incomplete and requires extensive discussion.

Lymph nodes play a pivotal role in the innate and adaptive immune response to natural antigens and vaccines. Lymphatic vessels direct lymph from the tissues to the lymph nodes scattered throughout the body. As lymph passes through the lymph node parenchyma, antigens come into contact with the effector cells of the adaptive immune system, initiating a cascade of immune processes that enable the recognition and neutralization of foreign antigens and pathogens [12]. Blood flows in and out of lymph nodes through the arterioles and venules of the hilum, respectively. Within the parenchyma, blood flows through postcapillary vessels (venous capillaries), so-called high endothelial venules (HEVs) lined with tall, cuboidal endothelial cells [12]. Immune cell migration to the lymph node in response to self or foreign antigens exposure relies on the coordinated functioning of adhesion molecules on the surface of leukocytes and venule endothelial cells. Such migration plays a fundamental role in regulating physiological processes, e.g., wound healing and angiogenesis, and pathological phenomena, e.g., inflammation and tumor cell filtration [13].

The number of lymph nodes in the human body ranges from 300 to 500, and their total weight is about 100 g. Studies have shown that the number of nodes decreases with age, and aging-associated degenerative features emerge in the lymph nodes, such as fibrosis, vitrification, lipomatosis, a reduction in the number of postcapillary vessels, and changes in the morphology and function of the specialized endothelial cells lining the venous capillaries [14]. Consequently, the amount of lymphoid tissue in the cortical and medullary zones of lymph nodes is reduced, as is the number and size of germinal centers in lymphoid follicles. These changes result in a reduced reactivity to antigen challenge. The number of follicular dendritic cells also decreases, and the ability to uptake and retain immune complexes is significantly impaired. These deficits result in decreased humoral immunity associated with impaired antibody production in the elderly [15] and an increased susceptibility to infections, one of the leading causes of morbidity and mortality in people over 65 [16]. Vaccination is an effective strategy to prevent the adverse health effects of infection; however, often the elderly do not generate long-term protective immunity [17,18,19,20]. Understanding the mechanisms underlying this problem is crucial for developing a new generation of vaccines and new vaccination strategies effective in aging individuals. Notably, accumulated fat deposits impair the ability of lymph nodes to filter cancer cells. The presence of hyaline deposits may also be associated with an increased risk of metastatic disease in the elderly [21,22]. 

Thus, aging-related lymph node structure disorganization and changes in the content of their immune cells seem to be major factors contributing to the aging of the immune system. This review discusses the current knowledge concerning age-related changes in lymph nodes and their impact on immune function.

## 2. Lymph Nodes Are Essential Components of the Immune System

The primary function of the lymph node is to coordinate the immune response to antigens transferred from the peripheral tissues. Thanks to their highly specialized architecture, lymph nodes act as filters that drain infected areas or filter out antigens from body fluids. To fulfill their roles in innate and acquired immune responses, they must attract and recruit leukocytes from the blood and peripheral tissues. There are two routes for cell influx into the lymph nodes: specialized blood vessels, i.e., HEVs lined with specialized high endothelial venule cells and afferent lymphatic vessels. During inflammation, lymphocyte accumulation in the draining lymph nodes significantly increases, while their exit via the draining lymphatic vessels is temporarily blocked. These two inflammation-induced mechanisms increase the likelihood of antigen capture by a fraction of T cells expressing the antigen-specific receptor (TCR) on their surface [23]. Lymph nodes are sites for lymphocyte maturation, activation, response to antigen challenge, homeostatic expansion, and tolerance induction [24,25]. In the lymph nodes, antigens absorbed by antigen-presenting cells, e.g., dendritic cells or macrophages, as well as present in a free form, are introduced to circulating naïve lymphocytes that migrate from the blood through the HEVs [25,26]. Upon activation, lymphocytes proliferate and develop effector functions and immune memory. With these acquired abilities for recognizing and clearing pathogens, T and B effector and memory lymphocytes patrol peripheral tissues through the bloodstream. These lymphocytes remain in circulation until recruited to sites of inflammation, where they facilitate an immediate, specific, adaptive, and local immune response, ensuring effective immune surveillance [27].

## 3. Aging-Related Structural, Cellular, and Functional Changes in Lymph Nodes

Lymph node degeneration is a widespread phenomenon, and its incidence increases with age [28]. Indeed, aging disrupts the architectural and cellular organization of lymph nodes [29,30,31,32,33]. In humans and rodents, aging lymph nodes lose cells and HEVs, and their size becomes smaller [34]. Adipocyte clusters and fibrosis are visible [34,35,36]. A series of histopathological sections of 161 lymph nodes from the head and neck of human cadavers of various ages showed variable degenerative processes that progressed with age [37]. Senile involution affected all functional zones of the lymph nodes, including the cortical and medullary zones. These changes, to some extent, explain some of the clinical conditions observed in the elderly, especially their decreased immune response to infections and increased risk of neoplastic metastases [37]. Old lymph nodes that drain the skin appear to be more altered than those that drain the mucosa [34]. Lymphocytes, like other immune cells, are essential for the proper functioning of lymph nodes. Therefore, their deficiency in the lymph nodes results in an inability of these structures to filter the lymphatic fluid of antigens and perform appropriate, rapid immune responses [28].

### 3.1. Stromal Cells

**Figure 1:**
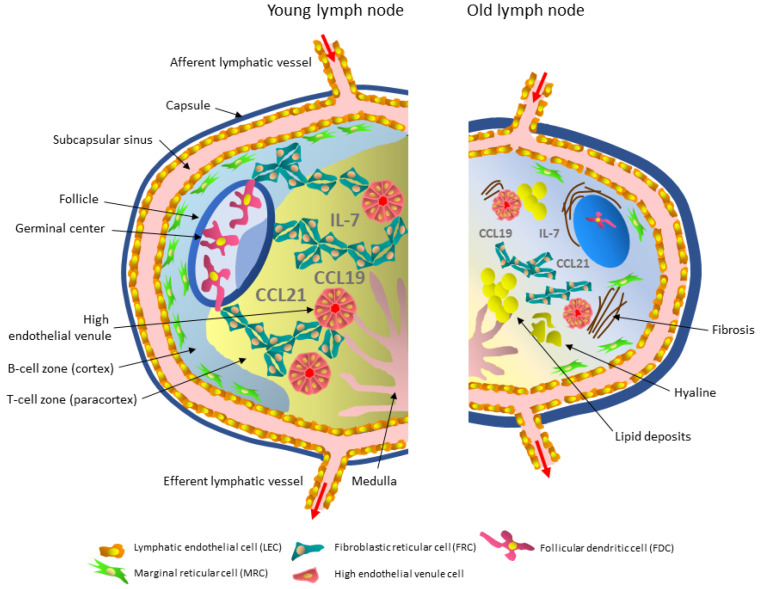
Simplified diagram of aging-associated lymph node stromal microenvironment changes. Compared to the young lymph node, the aged node is smaller, has a thick capsule, contains lipid and hyaline deposits, and is fibrotic. Functional zones become difficult to distinguish. Lymphatic vessels become permeable due to a loss of glycocalyx and GAP proteins. There are fewer HEVs, and high endothelial venule cells lining them are less rectangular and more compressed. The number of fibroblastic reticular cells (FRCs) is reduced, cells are more compacted, rigid, and form less “spongy” structures. Follicles are smaller with reduced or absent germinal centers and indistinguishable light and dark zones. The number of follicular dendritic cells (FDCs) is decreased, and the remaining cells are smaller. The number of marginal reticular cells (MRCs) is also reduced. The amount of homeostatic chemokines CCL19, CCL21, and IL-7 is decreased.

It was initially believed that stromal cells only had a role in the architectural organization of lymph nodes; however, it is now known that they are involved in some aspects of innate and adaptive immune responses [38,39,40]. Populations of nonhematopoietic stromal cells of mesenchymal origin provide the correct architecture and scaffolding necessary to direct cell movement in the functional zones of lymph nodes. Moreover, they facilitate antigen presentation to the circulating naïve T and B lymphocytes. Stromal cells create the appropriate microenvironment for immune homeostasis, produce and present chemokines that coordinate the movement of lymphocytes into and within the node, and activate and keep cells alive (Figure 1) [41,42].

The structural and functional disorganization of the lymph nodes that occurs with aging may adversely affect these processes. Histological evaluation of the structure of mesenteric lymph nodes in the elderly has revealed general fibrosis, thickening of the capsule and trabeculae, and increased amounts of connective tissue around the blood vessels [36]. The parenchyma of the nodes is typical of involuting lymphoid tissue [43,44,45,46,47,48]. Dense bundles of collagen fibers have been observed in the marginal sinuses of aging lymph nodes, and the sinuses are made of coarse-grained, fragmented fibers and fibroblasts. The reticular mesh of the sinuses is preserved. Lipomatosis affects both the cortical and medullary zones. Hyaline deposits are also observed [21,22]. The sinus system of the nodes involved in drainage and detoxification of the lymph is vague with irregular cell distribution. Moreover, connective tissue fibrosis observed during aging reduces lymph flow through the node, further affecting its function [45,49,50]. Thus, disorganization and dysfunction of the stromal cells seem to contribute to immunoaging (Figure 1, Table 1). The assessment of lymph node degeneration should be considered when planning medical interventions in the elderly, such as transplantation or desensitization to allergens [28]. However, there is still much to be explained about the influence of aging on the lymph node stroma.

#### 3.1.1. Lymphatic Endothelial Cells and Their Aging

Lymphatic vessels regulate the transport of tissue fluid and facilitate the absorption of macromolecules from peripheral tissues. Proper recirculation of fluids and cells via the effective transportation of lymph is required to maintain an organism’s homeostasis. Lymphatic vessels are also key routes of immune cell transport from tissues to regional lymph nodes during an immune response [51]. Under physiological conditions, the lymph contains over 1000 protein types and many other biomolecules, including a combination of plasma filtrate and soluble molecules produced by the peripheral tissues [52,53,54]. The lymph also carries insoluble material to the draining lymph nodes, including specific antigens, bacteria, and viruses. The concentrations of most of the transported endogenous and exogenous proteins are markedly decreased between the afferent and draining lymph, indicating they are effectively captured in the lymph nodes [55,56,57,58]. Lymphatic endothelial cells (LECs, Figure 1) line the sinuses of the lymph nodes, supply antigens from the tissues, and allow cells to move to other nodes. As a semipermeable barrier, LECs sort the lymph-borne antigens into the lymph node parenchyma and act as antigen-presenting cells. Leukocytes entering lymph nodes through the sinus system, and lymphocytes exiting the parenchyma migrate through the LEC layer. Lymphatic endothelial cells also participate in lymph-node organogenesis and bidirectional signaling with other sinus cells, e.g., antigen-presenting subcapsular sinus macrophages and dendritic cells, directing them to the proper nodal zone to create a unique lymph niche [59,60]. In addition, several animal models have demonstrated a direct role for LECs in mediating peripheral tolerance [61,62]. Lymphatic endothelial cells express immunosuppressive enzymes, e.g., indoleamine dioxygenase and inducible nitric oxide synthase, which inhibit dendritic cell maturation contributing to their immunosuppressive functions [63,64]. These endothelial cells may also support the activation of T lymphocytes by producing IL-7, which transmits pro-proliferative and antiapoptotic signals, and by long-term retention (archiving) of the antigen on their surface [65]. Thus, LECs act both directly as antigen-presenting cells and indirectly by modulating dendritic cells and T lymphocyte function and contributing to the maintenance of peripheral tolerance. Therefore, the dysfunction of LECs related to their role in regulating tolerance may contribute to the pathogenesis of autoimmune diseases [60].

Tissue swelling and an impaired response to pathogens are often observed in the elderly due to aging of the lymphatic system and altered lymph flow dynamics [66,67]. No differences in the number of LECs in the lymph nodes of old rodents were observed compared to young ones [15,68]. Still, an ultrastructural, biochemical, and functional comparative analysis of the lymphatic vessels of young adult and elderly mice showed a loss of extracellular matrix proteins with fewer and more scattered collagen bundles typically surrounding endothelial cells and a reduced number of smooth muscle cells in the vessels of aged animals. These changes reduced the contraction frequency and lymph flow velocity [69,70]. Consequently, the transport of pathogens, e.g., *Cryptococcus neoformans*, *Mycobacterium smegmatis,* and *Staphylococcus aureus* from the peripheral tissues to the draining lymph nodes, was impaired [70]. In older animals, the leaking of bacteria from the vessels into the surrounding tissue has been observed. Ultrastructural and proteomic analysis showed a decreased glycocalyx thickness in endothelial cells and a loss of GAP proteins. Oxidative stress has also been observed in older lymphatic vessels, resulting in increased permeability and a reduced ability to control tissue fluid homeostasis [70]. When challenged with influenza virus, the mesenteric lymph node LECs response in old mice is delayed, with delayed peak expansion, evidently due to impaired proliferation [68]. The decreased ability to transport bacteria to the draining lymph nodes and their tissue retention contributes to the reduced ability of the immune system to clear pathogens in the elderly. An aging-related increase in lymphatic vessel permeability (Figure 1) due to a reduction in glycocalyx and GAP connections may also affect the transport of large molecules, lipids, proteins, and products of tissue metabolism to the lymph nodes [71,72,73,74,75,76].

#### 3.1.2. High Endothelial Venule Cells and Their Aging

High endothelial venule cells are a critical subpopulation of endothelial cells lining the postcapillary HEVs (Figure 1) and act as a gateway for lymphocytes entering the lymph nodes and other secondary lymphoid organs in a multistage process of adhesion and extravasation involving chemokines, selectins, addressins, and integrins [25]. Thus, they enable recirculation (guidance) of naïve T and B lymphocytes and central memory cells in various lymphoid organs, ensuring effective immune surveillance. This migration is exceptionally efficient; an estimated five million lymphocytes migrate through this cell layer every second. Effective lymphocyte migration occurs thanks to the expression of CCL19 and CCL21 chemokines on HEVs, which interact with the CCR7 receptor present on the surface of lymphocytes [77]. High endothelial venule cells are characterized by a plump, almost cuboid shape and express peripheral lymph node vascular addressin (PNad) on their surface, which acts as a ligand for L-selectin and is a necessary component in the signaling and rolling of lymphocytes on the vessel surface [13]. Mouse studies have demonstrated the vital role of CD11c+ dendritic cells in controlling high endothelial venule cell phenotypes and functions. These lymphotoxin-expressing dendritic cells, and lymphotoxin itself, are indispensable for maintaining the mature high endothelial venule cell phenotype and HEV-mediated recruitment of lymphocytes in vivo, a process crucial to an effective immune response [78]. 

Analysis of aging-related changes in the nodal blood vessels showed a significant reduction in the number of HEVs in the paracortical zone of human lymph nodes and their degeneration level was substantial [28]. On the other hand, in the lymph nodes of old mice, the number of HEVs was unchanged, but there were morphological changes in the CD31+ endothelial cells, which were densely packed and squeezed (Figure 1). However, the consequences of this change were not explored further [15,31]. High endothelial venule cells in the mesenteric and inguinal lymph nodes of old mice were less rectangular and thinner than in young adult mice [68]. 

No significant age-related differences have been found in the expression of the ICAM-1 and PECAM-1 adhesion proteins on mouse endothelial cells [68]. However, there was impaired diapedesis of naïve T lymphocytes between high endothelial venule cells in the lymph nodes of aged mice and a decreased mobility of immune cells within the lymph node before antigen encounter.

Therefore, the decrease in the number of postcapillary vessels and changes in the morphology and functionality of high endothelial venule cells that occur with age reduce the migration of cells to aging nodes, contributing to their cellular disorganization. All these deficits make it difficult to initiate an early adaptive immune response and, thus, contribute to the susceptibility of the aged organism to infections [30].

#### 3.1.3. Marginal Reticular Cells and Their Aging

Marginal reticular cells (MRCs) represent a main element of the stroma. They are essential for the organization and function of secondary lymphoid organs (lymphoid tissue organizer) [79] and retain many of the characteristics of the cells responsible for organizing the lymphoid tissue [39]. It is suggested that MRCs could be converted to other stromal cell subsets, including follicular dendritic cells (FDCs) and fibroblastic reticular cells (FRCs) [79,80,81].

Marginal reticular cells surround the lymphoid follicles composed of B lymphocytes and fill the spaces between the follicles in the lymph node [79,80]. They are crucial for the integrity of the lymphatic endothelial cells in the subcapsular sinus by actively shaping the cellular microenvironment of the lymph node [82]. It is postulated that MRCs play an important role in transporting antigens to the lymphoid follicles [83] and supporting the survival and localization of innate lymphoid cells in the interfollicular niche by delivering IL-7 and other innate lymphoid cell survival factors [84,85]. Marginal reticular cells constitutively produce the CXCL13 chemokine and express mucosal addressin cell adhesion molecule 1 (MAdCAM-1) and other adhesion molecules. MAdCAM-1 is a ligand for receptors present on the surface of lymphocytes, e.g., α_4_β_7_ and α_4_β_1_ integrins and L-selectin, and thus, is involved in the homing of these cells to lymphoid tissues [85].

One hundred and twenty-two genes were differentially expressed between young and old adult MRCs [86]. Upon antigen challenge in young adult MRCs, the expression of as many as 923 genes changed compared to the resting state. In contrast, antigen challenge affected the expression of only 101 genes in aged MRCs [86]. It is then not surprising that the proliferative response of old marginal reticular cells is lower than young MRCs, and the capacity of old MRCs to become follicular dendritic cells or other stromal cells is decreased. Moreover, in aged mice, a reduction in the number of MAdCAM-1-expressing MRCs was observed in approximately 50% of lymph nodes [15]. Reducing the number of MAdCAM-1-expressing MRCs supposedly affects lymphocyte adhesion and migration, a phenomenon similar to that identified in old mice spleens [87]. Thus, MRC aging leads to impaired stromal cell expansion and a weakened response of follicle germinal centers, and an impaired humoral response [86].

#### 3.1.4. Follicular Dendritic Cells and Their Aging

Reticular cells producing the CXCL12 chemokine, marginal reticular cells being precursors of follicular dendritic cells and responsible for antigen transport, and follicular dendritic cells storing antigen and producing the CXCL13 chemokine re the stromal cells of follicle germinal centers [79,80,81,88,89,90,91] where the development of a B-cell-specific immune response occurs [92]. Follicular dendritic cells are specialized cells of mesenchymal origin containing long cytoplasmic protrusions, dendrites (Figure 1) [40,81,93,94]. FDCs do not enter the lymph node with the antigen but acquire it from other antigen-transporting cells. Their surface allows efficient capture and retention of large amounts of unprocessed, native antigens in the form of immune complexes (iccosomes) containing the antigen, antibodies, and opsonizing complement. Therefore, FDCs passively support the response of germinal centers to antigen challenge. Due to their extended lifespan, native antigens may be retained on their surface for months or years. Hence, it has been hypothesized that FDCs play a significant role in immune memory [95]. It is not yet known why the antigen is not internalized but remains on the cell surface. Follicular dendritic cells are present in the cortical zone of the lymph node and play a vital role in the organization of lymph nodes by secreting the CXCL13 chemokine, thereby attracting T and B lymphocytes expressing the CXCR5 receptor on their surface [96]. Moreover, FDCs are crucial for creating germinal centers [39], culminating in the production of high-affinity antibodies [96]. Follicular dendritic cells produce factors necessary for B-cell survival, including BAFF and APRIL [39]. The interaction of follicular T helper lymphocytes (Tfh CD4+) with B lymphocytes and FDCs in the germinal centers of the follicles provides adequate signals for activation, proliferation, somatic hypermutation, and maturation of B lymphocytes in the dark zone of lymphoid follicle germinal centers, thus facilitating a humoral response [97].

The aging of marginal reticular cells is responsible for defects in the MRC-to-FDC differentiation pathway [86]. Indeed, studies in mice have shown a reduction, both proportionally and in absolute terms, in the number of FDCs and their size in old lymph nodes compared to young nodes (Figure 1) [15,98]. In addition, the limited flexibility of the FDC network has been demonstrated [15]. These characteristics and, possibly, decreased Fc receptor expression impairs the acquisition and retention of immunocomplexes in the lymph nodes of old mice. The reduced size of the FDC network has a significant adverse effect on the cell counts in germinal centers, at 7 and 21 days post-immunization [86]. Furthermore, impaired antigen presentation to B lymphocytes and their impaired activation have been observed [99]. However, the efficient antigen presentation process can be restored by administering complement in vitro [15,99]. Nevertheless, old FDCs support a B-cell response in vitro to a lesser extent than young FDCs, even when cultured with T and B cells originating from young adult mice [100]. These data suggest that the age of the follicular dendritic cells in a node significantly affects the responsiveness of germinal centers to infection and immunization [86].

#### 3.1.5. Fibroblastic Reticular Cells and Their Aging

Fibroblastic reticular cells constitute the main subpopulation of lymph node stromal cells and play a significant role in the initiation, organization, and control of the adaptive functions of immune cells [40,101]. Fibroblastic reticular cells form a rigid, three-dimensional network useful for the migration, accumulation, and accommodation of lymphocytes inside the parenchyma of the node. They are highly reactive and plastic, which allows their activity to change within hours after vaccination and increase in number during the swelling of the lymph nodes typical of the first few days of an immune response [102].

Lymphotoxin β receptor (LTβR) expressed on FRCs is crucial for their survival and maintenance [103]. Lymphotoxin β is produced by dendritic cells, B cells, and T cells [104]. The bilateral relationship between T cells and FRCs mediated, among other things, by this protein, is critical for both cell populations [105]. When lymphotoxin β is depleted in young mice, lymph node organization becomes disturbed [104].

In reaction to an infection, FRCs lengthen and expand, increasing the available space for proliferating lymphocytes [102,106,107]. They form channels for the transport of small molecules and immune complexes. In addition, they direct the movement and position of dendritic cells, T lymphocytes, and antigens in the paracortical and medullary node zones by producing CCL19 and CCL21 chemokines [108,109,110]. In a state of immune homeostasis, CCL19 and IL-7 promote the survival of naïve T cells. Fibroblastic reticular cells have been identified in the B-cell (cortical) zone, T-cell (paracortical) zone, the medulla of the node, and tertiary lymphoid organs. Paracortical FRCs create niches for both T cells and dendritic cells, bringing them into physical contact and allowing MHC-independent activation of T cells. Another subset of FRCs creates niches for plasma cells in the medullary cords, promoting their proper localization and survival [111]. It has been shown in a mouse model that FRCs are necessary to initiate adaptive immunity to an influenza virus infection [112].

Fibroblastic reticular cells are the primary source of lymph node collagen, an essential component of the extracellular matrix [113]. At an appropriate thickness and abundance, collagen fibers determine the correct architecture and function of lymph nodes. However, an increased collagen fiber mass is often observed in some aging organs and is associated with fibrosis, significantly impairing their function [114]. Chronic inflammation caused by the presence of cytokines, such as TGFβ and IL-13 originating from, e.g., healing wounds [115], contributes to fibrosis in regional lymph nodes [4]. Similar processes along with an increased amount of proinflammatory cytokines are quite often observed in physiological aging. Such a similarity between inflammation-related and aging-related mechanisms was also observed with regard to FRCs [22,114].

A reduced number of FRCs and their simultaneous densification causes disorganization of the FRC network in old lymph nodes. An altered FRC architecture at the border of the cortical (B cells) and paracortical (T cells) zones in the mesenteric lymph nodes has been detected in aged mice. The clear boundary between these two functional zones has been blurred. As a consequence, the architecture of the lymphoid follicles is disturbed. The stroma of the cortical and paracortical zones appears more compressed and less reticulated [68].

During infection, FRCs numbers in old lymph nodes increases slightly and does not reach the level observed in young adult lymph nodes. The peak of FRC expansion is delayed in part because of their slower proliferation [68]. Node fibrosis can also contribute to a decrease in stromal cell proliferation [116]. In addition, old FRCs are less stretchy. These phenomena limit the lymph node’s ability to expand and accommodate the lymph draining the site of infection [117] and, thus, antigen presentation to lymphocytes and the influx of immune cells [118].

Fibroblastic reticular cells play an important role in maintaining the viability of naïve T cells by producing IL-7 and the CCL19 chemokine. Altered IL-7 expression by FRCs in aged mouse lymph nodes has been demonstrated and paralleled the decreased proliferation of the naïve lymphocyte population [31]. Moreover, FRCs of old mice produce fewer CCL19 and CCL21 homeostatic chemokines and are less sensitive to antigenic challenge than young adult mice cells [117]. The mechanism responsible for this phenomenon may be a decreased expression of heterotrimeric lymphotoxin β and its receptor [117,119]. Smaller amounts of CCL19 and CCL21 attract and support less naïve T lymphocytes, delaying and weakening the immune response [68].

Based on the above information, it can be assumed that the aging of FRCs significantly impairs the immune response to the antigen.

**Table 1:** Age-related changes in the structure and function of the lymph node stromal cells.

Cells	Young Lymph Node	Old Lymph Node
Lymphatic endothelial cells (LECs) 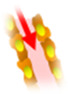	form lymphatic vessels and sinuses sort antigens present in the lymph and facilitate their transfer to the nodes produce chemokines that recruit innate immune cells to the lymph nodes upon antigen challenge, participate in lymph node expansion and contraction support the activation of T lymphocytes by producing IL-7	delayed proliferation upon immune stimuli loss of GAP proteins and reduced glycocalyx thickness in the lymphoid endothelial cell layer increased vessel permeability resulting in the impaired transport of bacteria, lipids, proteins, other large molecules, and products of tissue metabolism lower frequency of contractions and slow lymph flow, weakened control of tissue fluid homeostasis
High endothelial venule cells 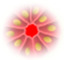	line the venous capillaries and act as a gateway for lymphocytes entering the lymph nodes produce CCL19 and CCL21 chemokines	reduced number of venous capillaries in the paracortical zone high endothelial venule cells are densely packed, less rectangular, thinner, and compressed disturbed diapedesis of naïve T lymphocytes between high endothelial venule cells
Marginal reticular cells (MRCs) 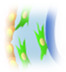	belong to the organizers of lymphoid tissue are crucial for the integrity of LECs in the subcapsular sinuses transport antigens to the follicles by secreting IL-7, affect the survival and localization of innate lymphoid cells in interfollicular niches are progenitors of follicular dendritic cells	reduced number lower proliferative response decreased capacity to become follicular dendritic cells, leading to impaired stromal cell expansion and the weak response of germinal centers to antigen challenge
Follicular dendritic cells (FDCs) 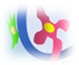	capture and retain large amounts of unprocessed, native antigens in the form of immune complexes containing the antigen, antibodies, and opsonizing complement participate in the organization of lymph nodes by secreting the CXCL13 chemokine, a ligand for CXCR5 receptor present on the surface of T and B lymphocytes are crucial for creating germinal centers produce factors necessary for B-cell survival interact with Tfh CD4+ and B cells	reduced number, size, and flexibility attenuated Fc receptor expression impairs the acquisition and retention of immunocomplexes in the nodes impaired antigen presentation to B cells leading to their impaired activation increased CXCL13 expression
Fibroblastic reticular cells (FRCs) 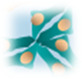	are the primary source of collagen form a three-dimensional scaffold necessary for the migration and accumulation of lymphocytes form channels for the transport of small molecules and immune complexes create niches for dendritic cells and T lymphocytes in the paracortical zone and plasma cells in the core zone by producing CCL19 and CCL21 chemokines, direct the movement of dendritic cells, T lymphocytes, and antigens in the cortical and paracortical zones secrete IL-7 for the survival of naïve T cells	reduced number of FRCs and their densification disrupts the FRCs network reduced extensibility and plasticity, resulting in a decreased ability of the node to increase the volume and accommodate the lymph flowing from the site of infection produce increased amounts of collagen, leading to the fibrosis of the node decreased production of CCL19, CCL21, and IL-7

### 3.2. Immune Cells

#### 3.2.1. T Lymphocytes in the Paracortex of the Lymph Node and Aging-Related Changes in the T Lymphocyte Microenvironment

**Figure 2:**
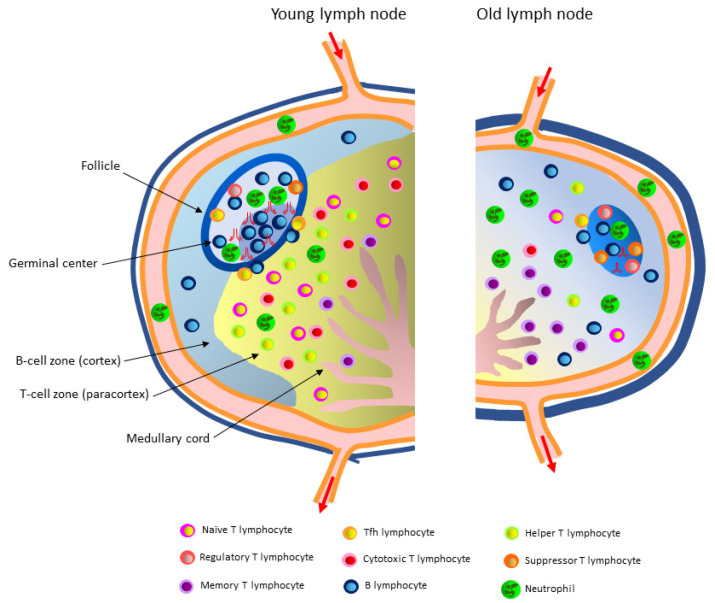
Simplified diagram of aging-associated lymphoid microenvironment changes. The number of immune cells in the old lymph node is reduced, and their functions are impaired. The boundary between the B-cell and T-cell zones is indistinguishable, and the medullary cords are thin. In small and germinal center-depleted follicles, a decrease in the number of proliferating B lymphocytes (CD20^hi^Ki67^hi^), Tfh lymphocytes (CD4+ PD1^hi^), monocytes (CD68^hi^), macrophages (CD163^hi^), and neutrophils (MPO^hi^) is observed, while the number of follicular regulatory T (CD4+ FoxP3^hi^) and suppressor T (CD4+ Lag3^hi^) lymphocytes is increased. Interactions between B cells and Tfh cells are disturbed. The follicle’s B lymphocytes produce less IgM and IgG antibodies which, in addition, have a reduced affinity for antigen. Outside the follicles, the number of naïve T lymphocytes (CD45RA+), cytotoxic T lymphocytes, and helper T lymphocytes is decreased, while the number of memory T lymphocytes (CD45RO+) is increased. An accumulation of old nonproliferating neutrophils is also observed.

Naïve lymphocytes constantly patrol the body in search of antigens and trigger a rapid immune response. They enter lymph nodes by a complex process beginning with a series of molecular interactions requiring the addressins expressed on the high endothelial venule cells, which are recognized by L-selectin (CD62L) expressed on lymphocytes [25]. Increased CCR7 receptor expression is a major determinant of the migration of effector and regulatory T cells to the afferent lymphatic vessels during inflammation [120]. The migration and survival of T cells are regulated by the system of stromal ducts composed of fibroblastic reticular cells. The presence of the CCL19 and CCL21 chemokines delimits the paracortical zone of the lymph node and increases the mobility of T lymphocytes [23,121]. Dendritic cells with an antigen on their surface form a mesh along which T lymphocytes move. T cells specific for a given antigen are retained in the paracortical zone of the lymph node and begin to proliferate intensively [122]. The contacts between antigen-presenting dendritic cells and antigen-specific T lymphocytes occur quickly and with great efficiency due to the high speed of T cell movement and the morphology of the dendritic cells, having an extensive system of antigen-presenting protrusions. Within one hour, between 500 and 5000 CD4+ T cells and approximately 500 CD8+ T cells can be observed near a single antigen-bound dendritic cell [123]. Upon recognizing the antigen, T cells are activated and express the CXCR5 surface receptor for the CXCL13 (BLC) chemokine secreted by the lymphoid follicle cells. Due to this chemotactic effect, the activated T cells leave the paracortical zone and move towards the follicle, where they interact with B lymphocytes [122] (Figure 2). 

Immunosenescence is associated with a gradual loss of naïve T cells and an increase in the number of memory and antigen-specific effector T cells that may eventually leave the lymph node and migrate to peripheral nonlymphoid organs. Studies in old rodents have shown disturbances in T lymphocyte movement, localization, and responses [30,31,124] (Figure 2). Reducing the size of the naïve T-cell pool negatively affects the immune response to new antigens appearing in the elderly body [125]. The reduced production of naïve T lymphocytes due to the thymic involution accompanying aging is additionally exacerbated by the unfriendly environment of secondary lymphoid organs, especially lymph nodes, which does not fully support their homeostasis [31]. Indeed, this reduction decreases the percentage and absolute number of naïve CD4+ and CD8+ T cells in the peripheral nodes of old mice [126]. Aging human lymph nodes also have a markedly reduced number of naïve CD45RA+ T cells [125]. In addition, the homeostatic proliferation of memory T cells is impaired in old mice; however, this phenomenon is less pronounced than for naïve T cells [31]. 

The surface area and volume of the cortical, paracortical, and medullary lymph node zones gradually decrease with age. The medullary cords become increasingly thinner, which is associated with a decrease in the T lymphocyte population [22,34]. Examination of the lymph nodes of middle-aged (66 ± 3 years) and age-advanced (88 ± 5 years) individuals showed a significant age-related reduction in cell density in all functional zones, including the paracortical layer. However, the relative and absolute small lymphocyte content in the paracortical (T cell) layer did not differ in an age-dependent manner. Immunohistochemical studies showed that CD4+ helper T cells, which regulate cellular and humoral immunity, are virtually absent in this zone in older study subjects [36]. On the other hand, an aging-related reduction in CD8+ T-cell counts occurs in the lymph nodes of older humans, along with an increased CD4+ to CD8+ T cell ratio [125]. With age, the percentage of fibroblasts increased proportionally in the paracortical layer, leading to the gradual replacement of lymphoid tissue with fibrous connective tissue [36]. A high content of plasma cells and eosinophils has been observed in the cords and medullary sinuses of lymph nodes originating from age-advanced individuals, which could reflect the development of autoimmune processes related to the decrease in the number of regulatory T lymphocytes [43,127].

The entry of naïve T cells into secondary lymphoid organs is a multistage process partly dependent on the interaction of the CCR7 receptor on their surface with the CCL19 and CCL21 chemokines expressed on the surface of high endothelial venule cells. In conjunction with CXCL13, these chemokines mediate the movement of lymphocytes in the lymph node and play an important role in directing T and B cells to the appropriate zones [31]. In the lymph nodes of old mice, impaired migration and T-cell survival occur due to decreased homeostatic chemokine levels and altered node architecture. In the same animal model, CCL19 is virtually absent, while the level of CXCL13 is significantly increased in aged nodes, resulting in preferential targeting of lymphocytes expressing receptors for CXCL13 to the lymphatic follicles instead of the paracortical zone [31,87]. Consequently, a significant number of CD3+ T cells can be observed in the B-cell zone; however, regardless of this, a substantial reduction in the density of CD3+ naïve T lymphocytes is observed in aged mice compared to young ones [31].

The importance of the lymph node microenvironment for T lymphocyte survival and function is highlighted by experiments involving parabiosis. Upon joining the bloodstream of young and old mice, T-cell circulation in both animals was unaffected; however, the total number of T cells in the old lymph nodes did not increase, despite the high T-cell influx from the young animal. In addition, stromal cell and T-cell cellularity in the lymph nodes of the young parabiosed animals decreased but was restored after the separation of the animals [128]. A decreased proliferation and activation of CD8+ T cells originating from the young mice and transferred to the old animals was observed in the lymph nodes of the recipients after immunization with influenza virus [68,129,130]. These results strongly suggest that aging-related changes in the lymph node microenvironment can be as important as changes in the T lymphocytes themselves for aging-related dyshomeostasis and dysfunction of the immune system.

In summary, changes in the lymphoid microenvironment with age, including deregulated steady-state and antigen-induced chemokine expression, contribute to decreased T-cell recruitment, limited access to factors necessary for survival, a disturbed distribution within the node resulting in limited access to antigens, and a reduced response to infection [124]. 

#### 3.2.2. B Lymphocytes in the Follicles of the Lymph Node and Aging-Related Changes in the B Lymphocyte Microenvironment

Generation of permanent protective immunity requires the production of long-lived plasma cells capable of switching the classes of synthesized antibodies with high affinity for the antigen [131]. In the germinal centers present in the cortical zone of lymph nodes, B cells, CD4+ T cells, follicular dendritic cells, and macrophages are connected by a network of stromal cells and cooperate with each other [88].

B lymphocytes expressing the CXCR5 receptor on their surface penetrate the lymph node mainly through postcapillary venules in the paracortical lymph node zone. They are then attracted to lymphoid follicles by CXCL13 produced by lymphoid follicle stromal cells, reticular cells, and dendritic cells [132]. After reaching the follicle, B lymphocytes form germinal centers. This process requires the presence of T helper cells. B lymphocytes proliferate after reaching the follicles and express the CCR7 receptor for the CCL19 and CCL21 chemokines produced in the thymus-dependent T-cell zone of the lymph node. This chemotactic interaction enables T and B lymphocytes to come closer to each other near the lymphoid follicle border and begin intensive cooperation in the formation of a humoral immune response [133].

During the primary B-cell response, antibodies with low antigen affinity are produced relatively quickly [134,135]. The first antibodies belong to the IgM class and play an important role in the opsonization of pathogens, induction of phagocytosis, and activation of the complement cascade. These functions and primary response rates play a crucial role in the protection against extracellular bacterial pathogens [136]. In addition, antibodies protect against viral infections by neutralizing viral particles and binding and blocking key proteins involved in cellular infection. Likewise, they can neutralize toxins. The later maturation of B-cell affinity, dependent on the presence of T cells, is slower but results in the production of highly specific antibodies [137] (Figure 2).

The development of an immune response in the lymphatic follicle germinal centers is a regulated, multifactorial process that requires spatial organization and the cooperation of stromal cells and cells directly involved in the innate and adaptive response [138]. The interactions between follicular T helper lymphocytes, B lymphocytes, and FDCs in the light zone of germinal centers provide adequate signals for the activation, proliferation, somatic hypermutation, and maturation of specific B-cell antigen affinity in the dark zone [97]. However, the clear boundary between the B-cell and T-cell zones in the nodes of aging rodents is blurred [68]. Similarly, significant differences in the nodes between young and old great apes have been observed. In aged animals, there is a marked reduction in the surface of the lymph nodes. The dark and light zones of germinal centers are largely indistinguishable in many old follicles [138]. In addition, mesenteric lymph nodes in aging humans have a small number of lymphoid follicles devoid of germinal centers along the cortical layer. The follicles are surrounded by ingrown, dense, fibrous connective tissue [36]. Compared to young individuals, there is a reduction in germinal centers’ size, number, and function in age-advanced individuals, also during viral and bacterial infections or after vaccination [127,139,140,141].

The formation of follicles is dependent on the CXCL13 chemokine and CXCR5 receptor expressed on B cells. In aging mice, a significant increase in CXCL13 protein secretion by marginal reticular cells and follicular dendritic cells and decreased CCL19 mRNA levels are observed, which is likely one reason for the disruption in the proper compartmentalization of lymph nodes [31]. Moreover, fourteen days after infection with the influenza virus, old mice lose the organized B-cell structure in the follicles of mesenteric lymph nodes [68]. 

A decreased number of CD20^hi^ Ki67^hi^ proliferating B cells and CD4+ PD1^hi^ Tfh cells and an increased number of follicular CD4+ FoxP3^hi^ and Lag3^hi^ suppressor T cells were observed in the germinal centers of the lymph nodes of old monkeys. These data suggest a disrupted reciprocal regulation between B and Tfh lymphocytes [138]. The ability of B lymphocytes to create germinal centers does not change significantly with age; however, a weakened Tfh lymphocyte response to antigen challenge both in aging humans and rodents negatively affects this process [31,142,143,144]. In old mice, poor communication between Tfh and B cells impairs the antigen-specific B cell response and effective selection of lymphocytes producing specific antibodies [97].

Follicular CD4+ regulatory T lymphocytes are located in the lymphoid follicle at the border of the B- and T-cell zones and control and limit the response of germinal centers to antigen [145,146]. Their increased number and, thus, suppressor activity in the follicles of old animals may likely reflect their increased circulating numbers [147]. The follicles of aged great apes contain fewer CD163^hi^ macrophages, myeloperoxidase-expressing neutrophils (MPO^hi^), and CD68^hi^ monocytes than those of young animals. Myeloperoxidase is involved in the production of hypochlorous acid, a chemical compound with strong bactericidal and antiviral properties [148]. Follicular CD68^hi^ monocytes and macrophages exhibit phagocytic functions and secrete IL-1β, CXCL9, CXCL10, and the ligands for CXCR3, a receptor highly expressed on T-cells present in germinal centers. These proinflammatory cells probably affect the activity of germinal centers by modulating the function and the local movement of B and T cells [138].

Such morphological and functional changes coincide and, in part, result from the disturbed structure of follicular dendritic cells, fibroblastic reticular cells, and other stromal cells, creating an unfavorable microenvironment [4].

The production of antigen-specific antibodies by memory B lymphocytes and plasma cells occurs in the germinal centers of the follicles [149]. In old mice, infection with West Nile virus is associated with impaired production of IgM and IgG antibodies [30]. Old rodents infected with the *Chikungunya* virus showed high antibody titers with a weak neutralizing function compared to the antibodies induced in young adult mice [150]. Defects in B lymphocyte functions are also detected in humans [151,152]. In the B lymphocytes of old individuals, the expression of the E47 transcription factor and activation-induced cytidine deaminase (AID) enzyme, responsible for class-switch recombination and somatic hypermutation, is decreased. These changes lead to the production of antibodies with reduced affinities for foreign antigens and a reduced capacity to fight infection or generate immunity after vaccination. Therefore, age-advanced individuals have reduced B-cell diversity, which correlates with their health status [153,154,155] (Figure 2).

#### 3.2.3. Neutrophils and Aging-Related Changes in Their Function

Neutrophils are the main effector cells of innate immunity against extracellular pathogens, acting through phagocytosis, the release of cytotoxic proteins, the production of reactive oxygen species, and the release of neutrophil extracellular traps. In addition to the desired anti-microbial effects, the long-term action of neutrophils can result in tissue damage, as observed in chronic inflammatory diseases. Similar side effects arising from the improper functioning of neutrophils can likely occur in the lymph nodes [156,157].

Neutrophils reach the lymph nodes by afferent lymphatic vessels and the bloodstream by HEVs through chemotaxis [158]. Neutrophil accumulation in the draining lymph nodes has been observed in mice after the administration of bacteria, e.g., *Staphylococcus aureus*, *Pseudomonas aeruginosa*, *Listeria monocytogenes*, *Yersinia pestis*, and BCG, infection with *Toxoplasma gondii* and *Leishmania major* [158,159,160,161,162,163,164], and intradermal administration of viruses such as modified vaccinia virus Ankara [164,165]. In mice, recruitment of neutrophils to the lymph nodes after tumor lysis has also been observed [166]. These observations show that different microbial, viral, or even sterile stimuli can contribute to the recruitment of neutrophils to the draining lymph nodes. When activated, these cells release granular proteins and chromatin, forming extracellular fibers that bind infesting microorganisms to prevent their spread [158]. However, in neutrophils from elderly human subjects and old mice the response to chemotactic signals is weaker than in cells from young individuals. This phenomenon might result from the constitutive activation of phosphoinositide 3-kinase [167,168]. Consequently, the rate of neutrophil exit from infected tissues and their migration into the nodes is slower in aged organisms, contributing to local tissue inflammation [169]. In addition, the phagocytic capabilities and apoptotic potential of neutrophils decrease in older humans and mice [170].

An increase in the percentage of mature, old neutrophils has been observed in the lymph nodes of healthy old mice compared to young adults (Figure 2). These cells do not proliferate and likely have delayed apoptosis. These data suggest that neutrophils from old mice live longer, which in turn increases their numbers in secondary lymphoid organs [171]. Moreover, the percentage of neutrophils expressing integrin CD11b and ICAM-1 in the lymph nodes of old mice is significantly increased. As these transmembrane glycoproteins are involved in the migration and activation of neutrophils, this increase suggests the occurrence of a chronic inflammatory state [171]. Neutrophils with high ICAM-1 expression and less susceptibility to apoptosis are also present in patients suffering from chronic inflammatory disorders [172], suggesting that such neutrophils present in aging individuals may contribute to an excessive inflammatory response. Furthermore, TGF-β levels expressed by neutrophils present in old nodes are increased, indicating that neutrophils infiltrating the secondary lymphoid organs of healthy old mice have altered functions that likely affect innate and adaptive immune responses [171].

An increased number of neutrophils invading the T- and B-cell zones in the absence of accompanying diseases has been observed in the lymphoid organs of old female mice [171]. Likely, this increase is a compensatory mechanism for their decreased effectiveness in fighting infection. Long-term neutrophilia during aging can lead to tissue damage, a persistent state of inflammation, and the increased risk of developing age-related diseases [171]. The great importance of neutrophils in older adults is exemplified by the increased morbidity and mortality observed when neutropenia or granulocyte defects are present [173,174,175,176].

The summary of aging-related changes in the lymph node immune cells is shown in Table 2.

## 4. Conclusions

Lymph nodes are secondary lymphoid organs that produce immune cells and respond immediately to foreign antigens, including vaccines. The structure of the lymph nodes ensures an efficient uptake, processing, and response to antigens present in the blood and lymph. This process aims to induce a long-term adaptive immune response in the host organism. The generation of permanent protective immunity requires the production of long-lived plasma cells secreting high-affinity class-switched antibodies [131]. These cells are the final product of tightly coordinated phenomena occurring in the germinal centers [177,178].

Aging causes progressive disruption of the lymph nodes as their number and size are reduced, and structure becomes disorganized. The most affected are the germinal centers [34,37,179]. The MRC-to-FDC differentiation pathway, a critical step in germinal center formation, is disrupted [86]. Moreover, the ability of follicular dendritic cells to uptake and retain antigen decreases [29,99]. The T helper lymphocyte response in germinal centers is also reduced [144,180], and their ability to support germinal center formation and B-cell selection is impaired [142,143]. The production of IgM and IgG antibodies by B lymphocytes decreases, and antibodies have a reduced affinity for antigens [153,154,155]. However, the aging of T and B lymphocytes is not the only factor contributing to the weakened response of germinal centers to antigen challenge. Equally important is the poor condition of the stromal cells constituting a scaffold over which immune cells migrate, defining zones for T and B lymphocytes and affecting immune cell homeostasis [112,181].

The importance of properly functioning lymph nodes in the course of severe infections was shown in COVID-19 patients. In those who did not survive the disease, the lymph nodes were devoid of germinal centers, follicular T helper lymphocytes did not differentiate properly, activated B cells were present outside of the germinal center microenvironment, and long-lived memory B cells with a high affinity for the antigen had not been produced [182]. Structurally and functionally, these changes resemble aging-associated changes, partly explaining a severe course of COVID-19 and a high risk of death due to this disease in older individuals. 

To sum up, immunosenescence leads to a weakened response to viral and bacterial infections and vaccines and an increased incidence of cancer and autoimmune diseases. The mechanisms responsible for the aging of lymph nodes have not been fully explained. Aging-related morphological and molecular changes in human and rodent lymph nodes affect the functioning of immune cells, which ultimately results in a diminished immune response. Understanding the molecular causes of aging-related changes can help identify new treatments to improve nodal health and, thus, improve immune responses and vaccine effectiveness in the elderly.

## Figures and Tables

**Table 2:** Summary of aging-related changes in the structure and function of lymphocytes and neutrophils in the lymph nodes.

Cells	Young Lymph Node	Old Lymph Node
T lymphocytes 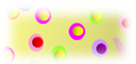	effective activation of T cells by antigens efficient proliferation production of effector cytokines migration to peripheral tissues to fulfill effector functions	impaired homeostatic proliferation of T cells disturbed movement, localization, and responses reduced size of the naïve T-cell pool including CD4+ and CD8+ T cells, increased memory T lymphocyte pool increased CD4+ to CD8+ T-cell ratio CD4+ T lymphocyte depletion in the paracortical zone decreased number of regulatory T-cells in the cords and medullary sinuses reduced number of CD4+ PD1^hi^ Tfh cells and increased number of CD4+ FoxP3^hi^ and Lag3^hi^ suppressor T cells in germinal centers
B lymphocytes 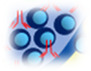	correct interactions between B lymphocytes, follicular T helper lymphocytes, and FDCs in germinal centers adequate activation, proliferation, somatic hypermutation, and maturation of antigen-specific B-cells effective production of antigen-specific antibodies	decreased number of CD20^hi^ Ki67^hi^ proliferating B cells in germinal centers loss of an organized B-cell structure in the follicles after immune stimulation disrupted reciprocal regulation between B and Tfh lymphocytes resulting in an impaired antigen-specific B-cell response and effective selection of lymphocytes producing specific antibodies impaired production of IgM and IgG antibodies decreased expression of the E47 transcription factor and activation-induced cytidine deaminase resulting in the production of antibodies with reduced affinity for foreign antigens weakened antibody neutralizing function
Neutrophils 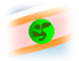	effective recruitment of neutrophils to the draining lymph nodes upon microbial, viral, or sterile stimuli search for and capture antigens release leukotriene B4 initiating the recruitment of other neutrophils from the vascular system upon activation, release granular proteins and chromatin, forming extracellular fibers capturing microorganisms to prevent their spread	increased number and percentage of not proliferating old neutrophils in the T- and B-cell zones of the lymph nodes weak response to chemotactic signals decreased apoptosis decreased phagocytosis increased percentage of neutrophils expressing integrin CD11b, ICAM-1, and TGF-β suggesting involvement in a chronic inflammatory state
